# Evolution of intermetallic GaPd_2_/SiO_2_ catalyst and optimization for methanol synthesis at ambient pressure

**DOI:** 10.1080/14686996.2019.1603886

**Published:** 2019-05-28

**Authors:** Elisabetta M. Fiordaliso, Irek Sharafutdinov, Hudson W. P. Carvalho, Jan Kehres, Jan-D. Grunwaldt, Ib Chorkendorff, Christian D. Damsgaard

**Affiliations:** a Center for Electron Nanoscopy, Technical University of Denmark, Lyngby, Denmark; b Department of Physics, Technical University of Denmark, Lyngby, Denmark; c Centro de Energia Nuclear na Agricultura, Universidade de São Paulo, Piracicaba, Brazil; d Institute for Chemical Technology and Polymer Chemistry, Karlsruhe Institute of Technology, Karlsruhe, Germany

**Keywords:** Methanol synthesis, CO_2_ hydrogenation, GaPd_2_, intermetallics, optimization, *in situ* XRD, *in situ* EXAFS, 50 Energy Materials, 205 Catalyst / Photocatalyst / Photosynthesis, 102 Porous / Nanoporous / Nanostructured materials

## Abstract

The CO_2_ hydrogenation to methanol is efficiently catalyzed at ambient pressure by nanodispersed intermetallic GaPd_2_/SiO_2_ catalysts prepared by incipient wetness impregnation. Here we optimize the catalyst in terms of metal content and reduction temperature in relation to its catalytic activity. We find that the intrinsic activity is higher for the GaPd_2_/SiO_2_ catalyst with a metal loading of 13 wt.% compared to catalysts with 23 wt.% and 7 wt.%, indicating that there is an optimum particle size for the reaction of around 8 nm. The highest catalytic activity is measured on catalysts reduced at 550°C. To unravel the formation of the active phase, we studied calcined GaPd_2_/SiO_2_ catalysts with 23 wt.% and 13 wt.% using a combination of *in situ* techniques: X-ray diffraction (XRD), X-ray absorption near edge fine structure (XANES) and extended X-ray absorption fine structure (EXAFS). We find that the catalyst with higher metal content reduces to metallic Pd in a mixture of H_2_/Ar at room temperature, while the catalyst with lower metal content retains a mixture of PdO and Pd up to 140°C. Both catalysts form the GaPd_2_ phase above 300°C, albeit the fraction of crystalline intermediate Pd nanoparticles of the catalyst with higher metal loading reduces at higher temperature. In the final state, the catalyst with higher metal loading contains a fraction of unalloyed metallic Pd, while the catalyst with lower metal loading is phase pure. We discuss the alloying mechanism leading to the catalyst active phase formation selecting three temperatures: 25°C, 320°C and 550°C.

## Graphical abstract

**Figure:**
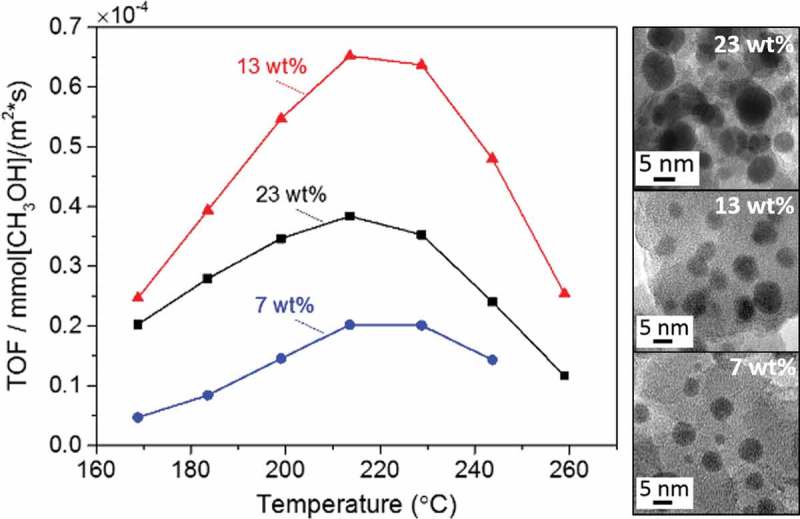

## 1. Introduction

The attention towards the utilization of intermetallic compounds for catalytic purposes has been constantly increasing in the last decade, due to their distinctive modified properties respect to those of the constituent elements [1]. The unique combination of covalent and ionic interactions in intermetallic compounds, as well as the presence of conducting electrons, results in attractive combinations of crystallographic and electronic structures for potential applications in catalysis and surface chemistry. For instance, Pt_3_Ti and PtPb intermetallic compounds are highly active for fuel electrocatalytic oxidation [2,3], whereas RuTi and NiSn are employed for the hydrogenation of unsaturated aldehydes [4,5]. Pd-Ga intermetallic compounds have also interesting catalytic properties for several reactions, such as selective semi-hydrogenation of acetylene [2], steam reforming of methanol [3], dimethyl ether production [4], and CO_2_ hydrogenation to methanol [5–12]. The last reaction, especially carried out at ambient pressure, has an important positive environmental impact since it can be performed using H_2_ derived from renewable power and involves CO_2_ sequestration, which is currently a global challenge.

Among the Pd-Ga family, the intermetallic Pd_2_Ga was reported to reach high activities in methanol synthesis from H_2_/CO_2_ feedstocks with a molar H_2_:CO_2_ ratio of 3:1 [9,12,13]. In particular, we have previously reported that Pd_2_Ga particles supported on high surface area SiO_2_, prepared by incipient wetness impregnation, is an active and selective catalyst for hydrogenation of CO_2_ to methanol at ambient pressure [9]. Moreover, this catalyst is very stable, in contrary to other novel intermetallic compounds, such as Ni_5_Ga_3_/SiO_2_, which suffers from deactivation upon catalytic testing [14,15]. We have reported that the activity of GaPd_2_/SiO_2_ was ~1.6 higher than the traditional Cu/ZnO/Al_2_O_3_ catalyst and, for consistent comparison with the Cu/ZnO/Al_2_O_3_ catalyst, we used a metal content of 23 wt.%. In this work, we focus on optimizing the GaPd_2_/SiO_2_ intermetallic catalyst in terms of metal content and reduction temperature in relation to its catalytic activity towards CO_2_ hydrogenation to methanol at ambient pressure. Moreover, we also study the catalyst structure and the formation of the active phase in a reducing atmosphere at increasing temperature. We use a powerful combination of complementary techniques, such as *in situ* X-ray diffraction (XRD), X-ray absorption near edge fine structure (XANES) and extended X-ray absorption fine structure (EXAFS) to reveal the formation mechanism of the active GaPd_2_ intermetallic phase at ambient pressure. We discuss the alloying mechanism leading to the active phase formation at three temperatures, namely 25°C, 320°C and 550°C.

## 2. Experimental details

### 2.1 Catalyst preparation and testing

Intermetallic GaPd_2_/SiO_2_ catalysts with 23 wt.%, 13 wt.% and 7 wt.% metal loadings were prepared by incipient wetness impregnation of Pd(NO_3_)_2_ (Carl Roth, Germany) and Ga(NO_3_)_3_ (Sigma Aldrich, USA, 99.9%), dissolved in a 4M HNO_3_ solution, into high surface area SiO_2_ (241m^2^/g, Saint Gobain Norpro, France). The amount of catalyst used was 0.12 g, 0.06 g and 0.03 g for the 23 wt.%, 13 wt.% and 7 wt.% metal loadings, respectively.

The catalyst preparation and testing scheme is adapted from Ref [9]. The catalyst precursors were dried and calcined under static air at 120°C and 260°C, respectively. Then, the precursors were reduced in a flow of 25% H_2_/Ar at ambient pressure at four temperatures, namely 400°C, 500°C, 550°C and 600°C. Finally, the temperature was decreased to 165°C for catalytic testing and direct comparison of the catalysts with the three different metal contents.

Activity measurements were performed at ambient pressure in a quartz glass reactor (d_i_ = 6 mm) with a catalyst sieve fraction of 0.212–0.354 mm. Total flow rate of the stoichiometric feed gas, CO_2_ (25%) and H_2_ (75%), was 100 N ml/min, while the catalytic bed volume was 1.13 cm^3^. The temperature gradient across the reaction zone was monitored by two thermocouples placed before and after the catalyst bed. Agilent 7890A gas chromatograph (GC), equipped with a thermal conductivity detector (TCD) and flame ionization detector (FID), was used to analyze the reaction mixture. The configuration of the GC is described in detail elsewhere [15]. A product gas was injected every 15 min and 5 measurements were carried out at each temperature to ensure stable reading.

### 2.2 In situ XRD

The *in situ* XRD experiments were performed at the I711 beamline, MAXLAB, Sweden. The optical components at beamline I711 is a vertical focusing mirror and a single bounce Si-based monochromator [16]. The sample stage at I711 is equipped with a Newport 4-cirle diffractometer with a kappa geometry and an Agilent Titan CCD detector with a 165 mm diameter. Details about the design of the *in situ* cell can be found elsewhere [17]. The catalyst is placed in a 1 mm sapphire capillary with an inner diameter of 0.8 mm. Sample heating is performed with a custom designed heater setup, consisting of an Inconel® block, accommodating three heating cartridges of the type KMFE0035A004A, Firerod® from Watlow, with an overall power of 120 W. The opening of the heater permits the observation of scattering patterns in an angular range of 2ϴ = 0–65°.

The temperature is regulated by a combination of a Eurotherm 2416 PID controller and a solid state relay [18], the temperature for the feedback loop is measured about 1–2 mm downstream from the end of the catalyst bed with a 0.5 mm NiCr-Ni thermocouple, inserted inside the sapphire capillary. The heater mount consists of a stainless steel plate, guided by two metal rods that are installed in a metal bracket, heat transfer from the Inconel® block to the mounting is minimized by a spacer from porous isolation material.

Heat dissipation during the experiments is suppressed with a custom-made isolation from ceramic fiber board of the type 1400c, including 10 µm thick entrance and exit windows from scratch free mica. The *in situ* cell is connected to a gas system including 4 mass flow controllers a pressure controller, permitting investigations of the catalyst materials at flows between 0.03 and about 100 ml/min and pressures between 100 mbar and 10 bar.

### 2.3 In situ XANES and EXAFS

*In situ* XANES and EXAFS spectra were acquired in transmission mode at SAMBA beamline, Soleil Synchrotron facility, France. The data were acquired using the Quick-EXAFS edge jump setup [19] and the X-rays were monochromatized by channel-cut monochromators: Si(111) was used for Ga-K edge (10,367 eV) and Si(311) for Pd-K edge (24,350 eV).

The calcined catalyst precursor was loaded in a quartz capillary (d_i_ = 1.0 mm, wall thickness 0.02 mm). The reduction was carried out in a 25% H_2_/He gas mixture flowing at 50 mL min^−1^, the reactor was heated by an Oxford gas blower from room temperature up to 550°C at the rate of 5°C/min, while the temperature was measured by a thermocouple placed directly the capillary [20]. The XAS spectra were continuously recorded at frequency of 1 Hz. To improve the signal-to-noise ratio 58 spectra (up and down movements of the scanning) were merged, corresponding to nearly 30 s of measurement of 2.5°C.

The data analysis was performed using Athena and Artemis software of the IFEFFIT package [21]. The spectra were energy calibrated using Pd and W metal foils as references. The X-ray absorption spectra were normalized and background subtracted. Then the EXAFS function χ(k) was extracted and the k^2^-weighted χ(k) Fourier-transformed. The theoretical amplitude functions and phase shifts for Pd and Ga absorbers were calculated using the atomic positions given by crystallographic data [22] and by building an atomic cluster using the FEFF6.0 code for Artemis [23]. The number of neighbors (N), atomic distances (R) and mean square deviation of interatomic distances (σ^2^), were determined through the refinement of the theoretically calculated spectra with the experimental spectra. The energy misalignment between the theory and experiment was compensated by the energy shift factor (ΔE_0_). The amplitude reduction factor (S_0_^2^) for Pd was obtained by refining the spectrum of a Pd foil, whereas that of Ga was determined from the spectrum of Ga(NO_3_)_3_. The fit quality is represented by *ρ*-factor. The curve fitting of the k^2^-weighted Fourier Transformed spectra for Pd and Ga was carried out simultaneously using a k range of 2–13 Å^−1^ and an R range of 1–3 Å.

### 2.4 Transmission electron microscopy

TEM images of the reduced intermetallic catalysts dispersed on a Cu/C TEM grid were acquired using a FEI Tecnai T20 TEM microscope, operating at 200 kV. The TEM images were used to measure the size distribution of the catalysts with the three different metal content. The standard error of the mean is defined as standard deviation.

## 3. Results

### 3.1 Optimization of metal content

In our previous work [9], we have shown that intermetallic GaPd_2_/SiO_2_ catalyst prepared by wetness impregnation of the metal nitrates is active, selective and stable for ambient pressure CO_2_ hydrogenation to form methanol. Here, we further optimize the catalyst in terms of metal content by investigating the intrinsic catalytic activity and selectivity of the GaPd_2_/SiO_2_ catalyst with three metal loadings, namely 23 wt.%, 13 wt.% and 7 wt.%.

**Figure 1.**
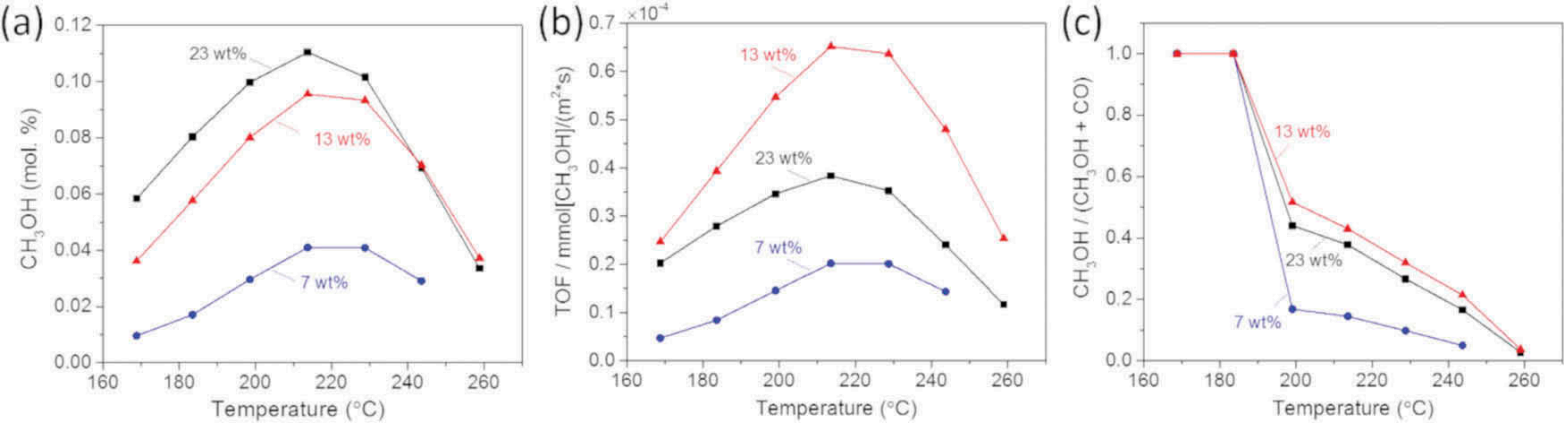
(a) Methanol yield, (b) Turnover frequency (TOF) and (c) CH_3_OH-to-CO ratio measured from the GaPd_2_/SiO_2_ catalyst with the three metal loadings, as a function of reaction temperature.

Figure 1(a) shows the methanol yield plotted as a function of the reaction temperature measured from the three catalysts. We find a decrease in the activity with decreased metal content and the methanol yield is significantly lower for the catalyst with 7 wt.% metal content compared to the other two with higher metal content. In order to find the optimum catalyst in terms of active surface area, we calculated the turnover frequency (TOF) values. Figure 1(b) shows the TOF values corresponding to methanol production at atmospheric pressure of the GaPd_2_/SiO_2_ catalyst with the three metal loadings, as a function of the reaction temperature.

**Figure 2.**
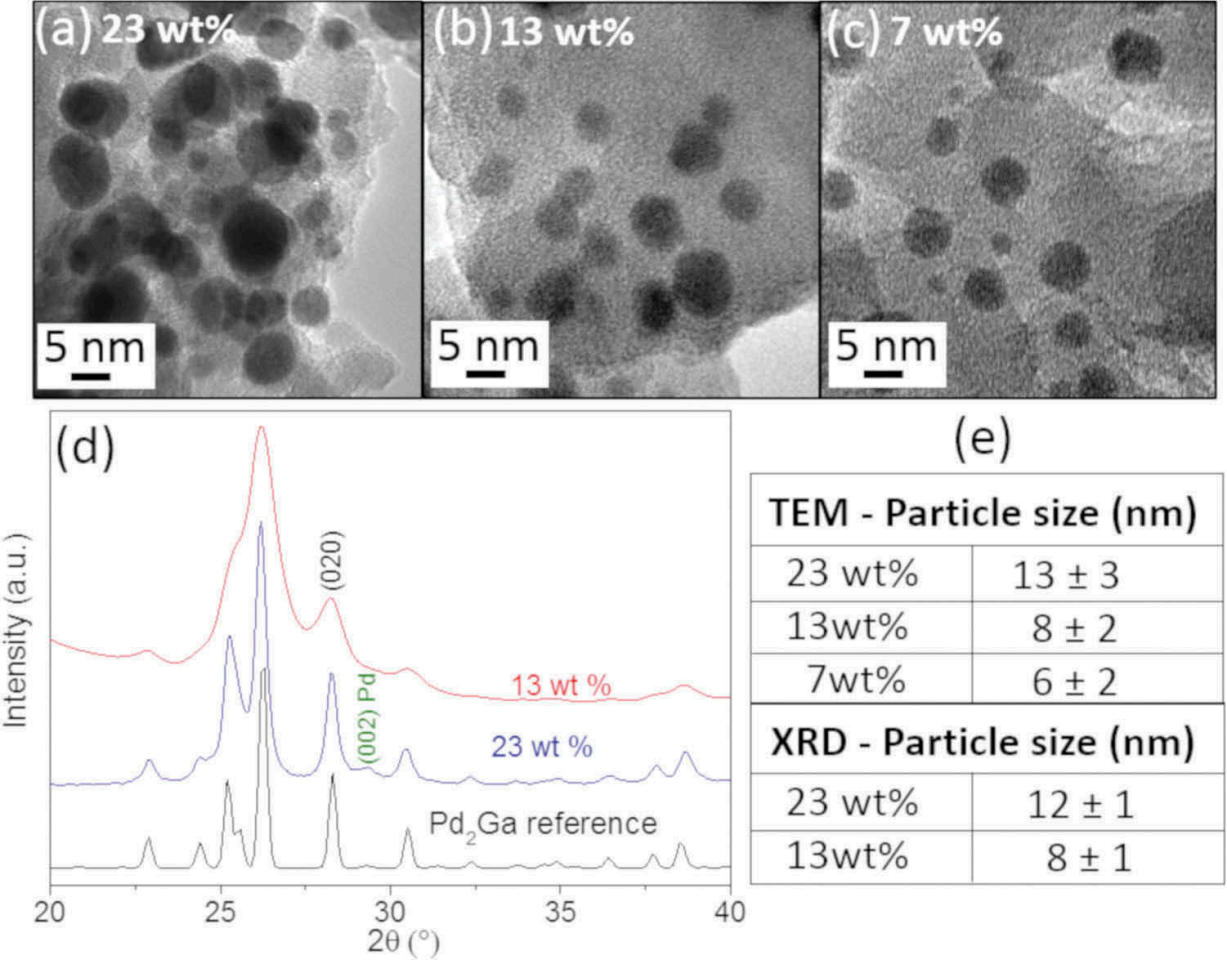
TEM images corresponding to the GaPd_2_/SiO_2_ catalyst with (a) 23 wt.%, (b) 13 wt.% and (c) 7 wt.% metal loadings after reduction at 550°C and catalytic testing. (d) *In situ* XRD patterns of the catalyst with 23 and 13 wt.% metal loadings after reduction at 550°C and catalytic testing. The reference for the GaPd_2_ phase is also shown (GaPd_2_ (#ICSD 409,939). (e) Estimated average particle size from TEM images and *in situ* XRD patterns.

In detail, the TOF values (in units of mol_CH3OH_/m^2^·s) were calculated based on the surface area of the nanoparticles. The average particle size was estimated from a collection of TEM images assuming spherical particles and by Scherrer broadening analysis of the crystallographic reflections (020) of the *in situ* XRD patterns assuming single crystalline particles. Figure 2(a-c) shows examples of TEM images acquired after reduction at 550°C and catalytic testing of the three catalyst metal loadings, showing smaller particle size and higher dispersion with decreasing metal content. *In situ* XRD patterns were acquired only from the catalyst with 23 wt.% and 13 wt.% metal content after catalytic test and they are reported in Figure 2(d). A table containing the average values for particle size is reported in Figure 2(e), which shows a good agreement between TEM and XRD results.

Figure 1(b) shows that the GaPd_2_/SiO_2_ catalyst with the 13 wt.% metal loading has the highest intrinsic activity among the three tested catalysts, with corresponding average particle size of 8 nm. The intrinsic activity, at temperature close to equilibrium, for the 13 wt.% metal loading catalyst is 3 and 2 times higher than the 7 wt.% and 23 wt.% metal loading GaPd_2_/SiO_2_ catalysts, respectively. Moreover, it is more than 3 times higher than that of the traditional Cu/ZnO/Al_2_O_3_ catalysts as previously reported [9]. Finally, our best measured intrinsic activity is comparable to values recently reported from optimized GaPd catalysts prepared via a colloidal synthesis route [10].

The only by-product formed during the reaction is CO, which is also the case for the traditional Cu/ZnO/Al_2_O_3_ catalyst. Figure 1(c) shows the selectivity toward CH_3_OH compared to CO of the three catalysts, as a function of the reaction temperature. We find that the selectivity is highest for the GaPd_2_/SiO_2_ catalysts with 13 wt.% metal content, which is more than 2 times higher than the catalyst with 7 wt.% of metal content and slightly better than the catalyst with 23 wt.% metal content.

The catalytic results demonstrate that the intermetallic GaPd_2_/SiO_2_ catalyst with 13 wt.% metal loading prepared by simple impregnation of metal nitrates is a remarkable candidate for CO_2_ hydrogenation to methanol at ambient pressure. They also indicate a particle size dependence both in the TOF and selectivity during CO_2_ hydrogenation to methanol reaction.

### 3.2 Formation of the active phase by in situ XRD, XANES and EXAFS

In the following, we focus on investigating the active phase formation of the GaPd_2_/SiO_2_ catalyst with 23 and 13 wt.% metal loadings. We do not further investigate the catalyst with 7 wt.% metal content due to its poor activity and selectivity compared to the catalysts with higher metal loadings.

Temperature programmed reaction (TPR) measurements were carried out at synchrotron radiation sources, where *in situ* XRD, XANES and EXAFS spectra were recorded as a function of the increasing temperature. *In situ* XANES and EXAFS measurements were necessary due to the amorphous nature of the Ga species, which cannot be detected by XRD measurements.

**Figure 3.**
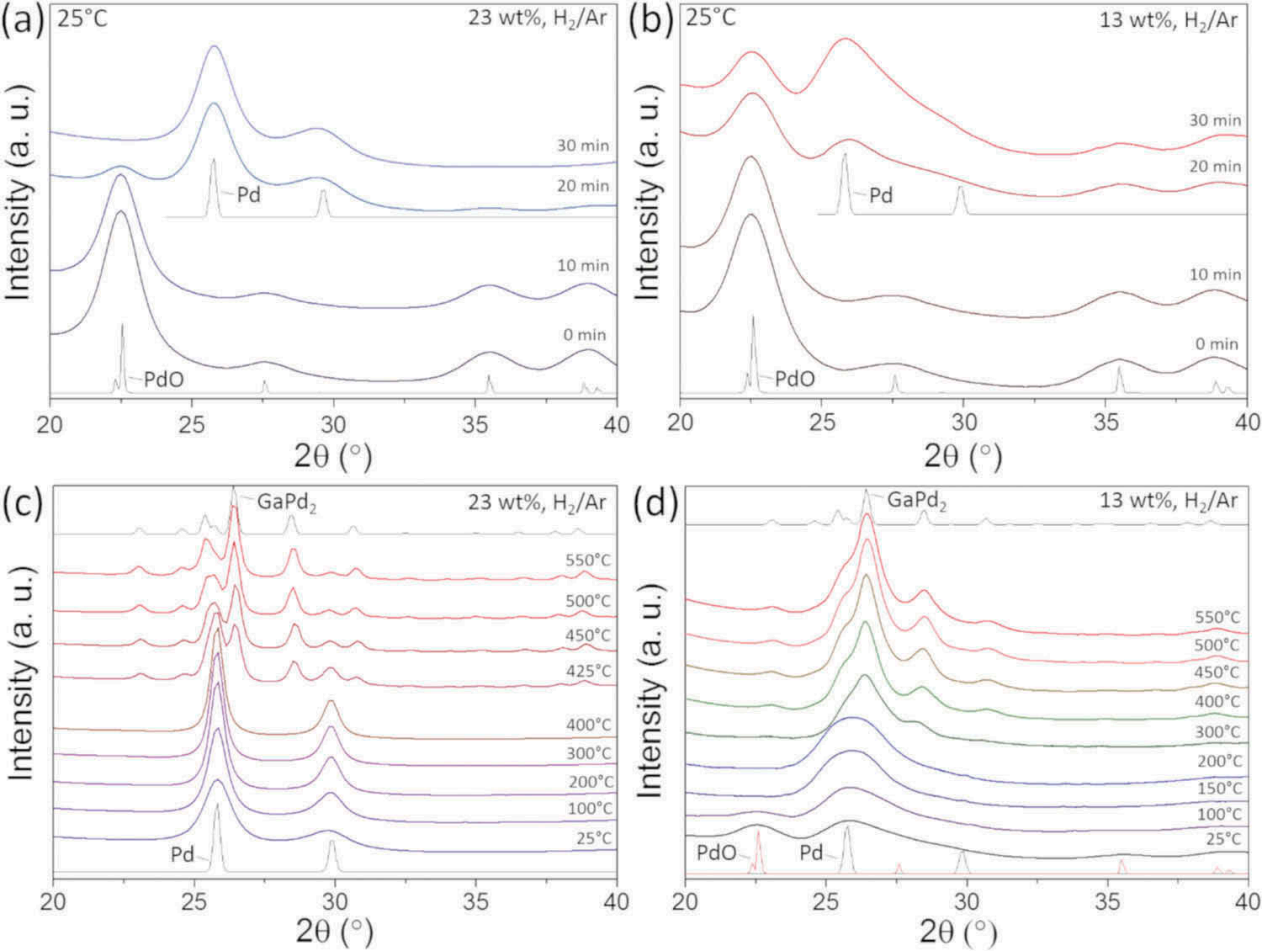
(a) *In situ* XRD patterns in a mixture of 25% H_2_/Ar acquired at room temperature from the calcined catalyst with (a) 23 wt.% and (b) 13 wt.% metal loading. TPR in a mixture of 25% H_2_/Ar acquired from the catalyst with (c) 23 wt.% and (d) 13 wt.% metal loading. For guidance, the data are plotted together with the simulated XRD patterns (thin lines) for the three main sample phases occurring during the reduction process Pd (#ICSD 52,251), PdO (#ICSD 24,692) and GaPd_2_ (#ICSD 409,939).

Prior to the TPR measurements, the catalysts were dried at 100°C for 3 h and calcined at 260°C for 2 h. Figure 3(a,b) shows the *in situ* XRD patterns recorded within 30 min at room temperature in a mixture of 25% H_2_/Ar from the calcined catalysts with 23 and 13 wt.% metal loadings, respectively. We observe that in the case of the catalyst with the higher metal content the PdO phase is fully reduced to metallic Pd at room temperature, whereas the catalyst with the lower metal content maintains a mixture of PdO and Pd under the same conditions.

This can be explained by considering that in the higher loaded Pd-catalyst, the Pd atoms naturally lie closer together. Indeed, it has been observed earlier that well dispersed PdO is more difficult to reduce than PdO that is present as particles [24,25].

The XRD patterns of the catalysts with 23 and 13 wt.% metal loadings during TPR are reported in Figure 3(c,d), respectively. The temperature was increased by 5°C/min and the total reduction time was 2 h. Figure 3(c) shows that the catalyst with the higher metal loading retains the metallic Pd phase from room temperature to around 400°C. In this temperature range, we observe a sintering of the Pd phase, as indicated by the sharpening of the reflections with increasing temperature. At 400°C metallic Pd particles start alloying with gallium and the formation of the GaPd_2_ phase is completed at around 500°C. The TPR is terminated at 550°C. An estimate of ~9 wt.% metallic Pd was determined by comparison of the intensities of GaPd_2_ and Pd reflections, after completion of TPR. Using the Scherrer equation the average Pd particle size was estimated as 14.5 nm.

Figure 3(d) shows the TPR of the catalyst with the 13 wt.% metal loading, which is different in a few aspects compared to the TPR of the catalyst with the higher metal content. At room temperature, we observe a mixture of PdO and Pd phases. The reduction from PdO to metallic Pd is completed at 150°C. Broad reflections are measured at this temperature, indicating the presence of smaller Pd crystallites compared to the catalysts with the 23 wt.% metal loading. The small Pd nanocrystals start alloying with gallium at 300°C, and the formation of the GaPd_2_ phase is completed at 500°C. The TPR is terminated at 550°C. No metallic Pd is observed for 13 wt.%.

The absence of Ga-compounds in the XRD patterns indicates the presence of highly dispersed noncrystalline Ga_2_O_3_ species upon calcination, which are further reduced on the surface of the Pd at elevated temperatures. *In situ* XANES and EXAFS measurement are therefore needed to elucidate the evolution of the noncrystalline Ga species.

**Figure 4.**
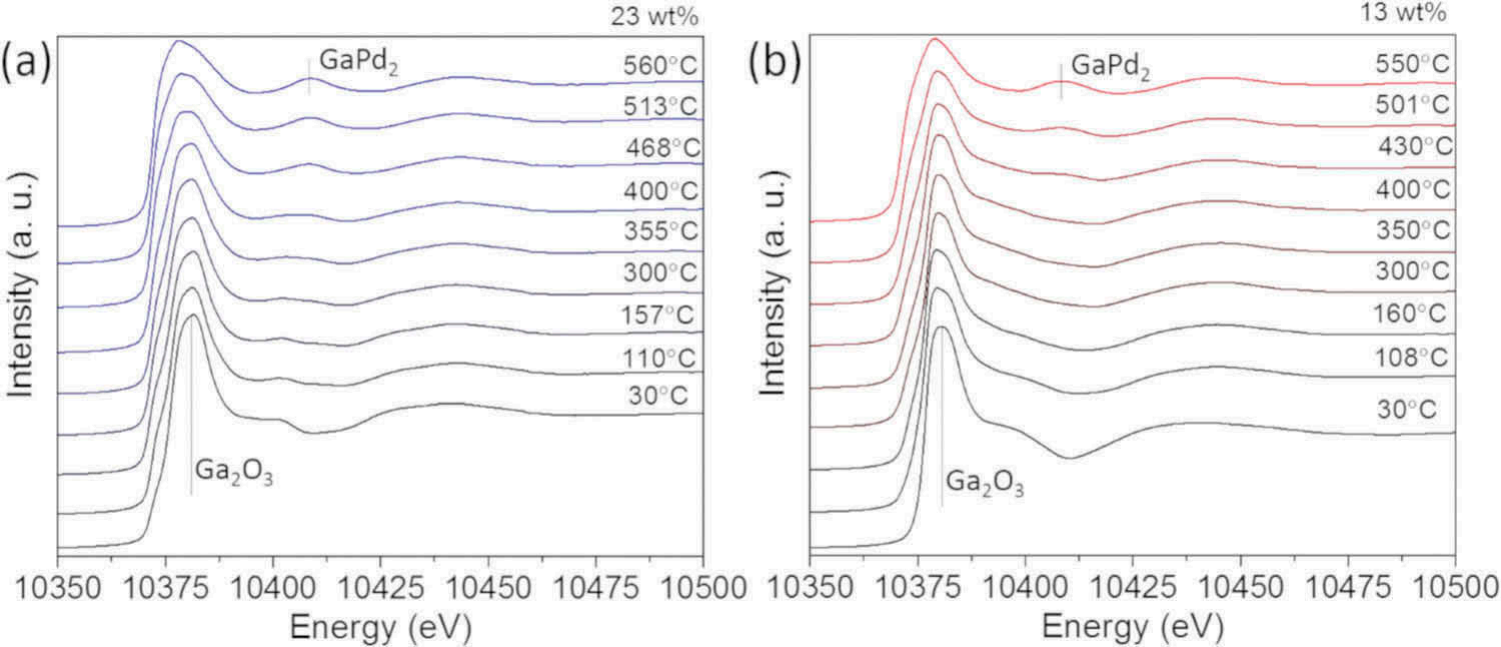
(a) *In situ* XANES spectra in a mixture of 25% H_2_/Ar acquired at increasing temperature from the calcined catalyst with (a) 23 wt.% and (b) 13 wt.% metal loading.

Figure 4(a,b) show *in situ* XANES spectra for the Ga-K edge of the catalysts with 23 and 13 wt.% metal loadings, respectively, acquired at increasing temperature in a mixture of 25% H_2_/Ar from calcined precursors. The evolution of the Ga-K edge shows a similar behavior in both catalysts: at room temperature a main peak is measured at 10.381 eV, indicating that Ga is in an oxide phase with tetrahedral coordination, which corresponds to Ga_2_O_3_. The Ga species reduce with increasing temperature, but no transition to pure metallic Ga species is measured for both catalysts. The alloying starts above 300°C, and the GaPd_2_ phase formation is completed at 500°C, which is in good agreement with the *in situ* XRD results.

**Figure 5.**
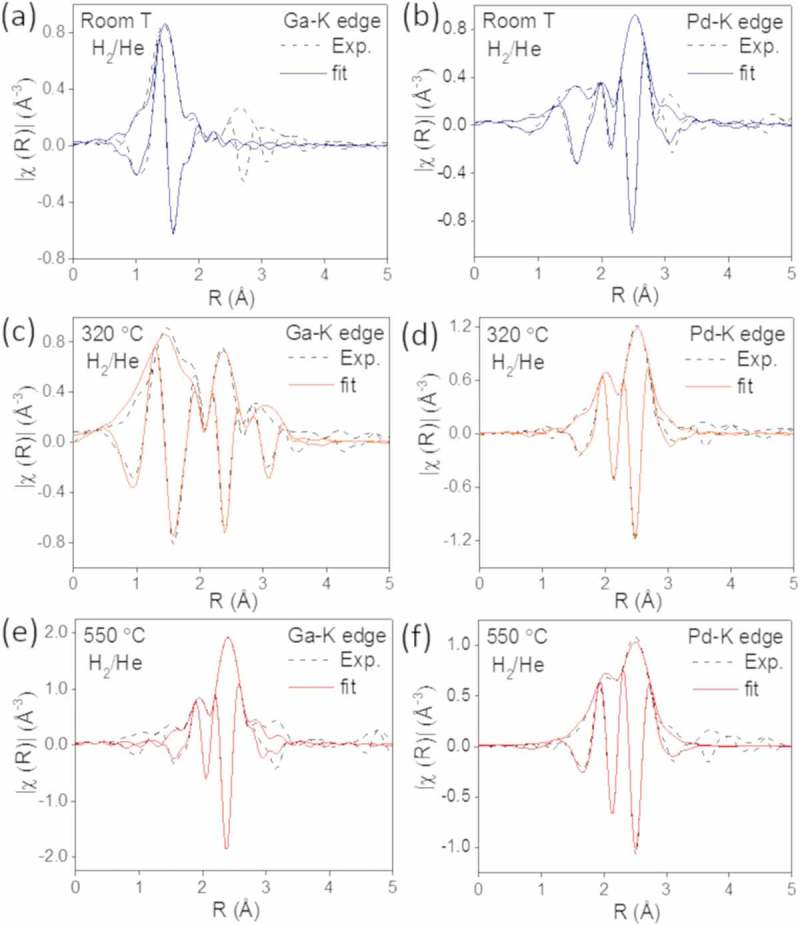
Experimental (dashed lines) and fitted (solid lines) Fourier transformed EXAFS spectra from the catalyst with 23 wt.% metal loading collected in H_2_/He at the Ga-K edge at (a) room temperature, (c) 320°C and (e) 550°C and at the Pd-K edge at (b) room temperature, (d) 320°C and (f) 550°C.

While XANES describes the local geometry, type of atoms and oxidation state of the intermetallic catalysts, EXAFS captures the local structure and complements the knowledge gained from the XRD results. Figure 5 shows the experimental and adjusted Fourier transform of the k^2^-weighted EXAFS spectra collected in a 25% H_2_/He mixture at the Pd-K edge and Ga-K edge from the GaPd_2_/SiO_2_ catalyst with 23 wt.% metal content at three temperatures: 25°C, 320°C and 550°C. The structural parameters obtained from the fitting procedures are presented in Table 1. The experimental and fitted data for GaPd_2_ crystal structure are in good agreement, as the ρ factor is within the acceptable range [26]. Figure 5(a,b) show EXAFS spectra acquired at 25°C in a 25% H_2_/He mixture for the Ga-edge and Pd-edge, respectively. The Ga and the Pd atoms are situated in two different sites: the Ga atoms are surrounded by O atoms, indicating that the Ga is in an oxidic state. The fitting required the inclusion of two O distances, which suggests a distorted oxide structure. The Pd atoms are surrounded by O atoms, but a fraction of metallic Pd was observed both at the first coordination shell. Thus, PdO was partially reduced at room temperature. The intensity ratio between the Pd-Pd peak and the Pd-O peak and particularly the high Pd-Pd coordination number with typical values for bulk Pd of 2.76 Å, indicates a dominating metallic Pd phase over a PdO phase at room temperature in the presence of H_2_. This correlates with results from XRD shown in Figure 3(a). Figure 5(c) and (d) show EXAFS spectra acquired at 320°C in a 25% H_2_/He mixture for the Ga-edge and Pd-edge, respectively. We find that the Ga atoms have three shells of neighbors, namely O, Pd and Ga atoms, while the Pd atoms are surrounded by Ga and Pd atoms. The results indicate that at this intermediate temperature a mixture of metallic Pd and Ga oxide coexists with a Pd-Ga compound. This observation correlates with the XANES. In contrast, XRD analysis shows no crystalline GaPd structure below 400°C, i.e. it remains rather X-ray amorphous. This be explained by either very small crystallites or by an amorphous nature of the GaPd at this intermediate state. Figure 5(e,f) show EXAFS spectra acquired at 550°C in a 25% H_2_/He mixture for the Ga-edge and Pd-edge, respectively. At this temperature, the Ga atoms are only surrounded by Pd atoms and the Pd atoms are surrounded by Pd and Ga atoms, supporting the formation of the GaPd_2_ phase.

**Table 1.** Structural parameters obtained from EXAFS data analysis at Pd and Ga-K edges^a^ acquired from the catalyst with 23 wt.% metal content. Pd S_0_^2^ = 0.83 and Ga S_0_^2^ = 0.88. Pd-Pd atomic distance refined for Pd foil was 2.74 Å. ^b^Adjusted parameter. ^c^Constrained parameter. ^d^10% correction was applied on the basis of XRD results that showed that part of the Pd atoms were not alloyed.

23 wt.%	Edge	Shell	Atom	*N*	*R*(Å)	*σ*^2^(10^–3^ Å^2^)	ΔE_0_ (eV)	*ρ* (%)
RT H_2_/He	Pd-K	1^st^	O	4^c^	2.01 ± 0.01^b^	2.5 ± 0.5^b^	3.5 ± 1.2	3.1
		2^nd^	Pd	6.8 ± 1.2^b^	2.76 ± 0.01^b^	9.8 ± 1.6^b^		
	Ga-K	1^st^	O	2.7 ± 0.3^b^	1.86 ± 0.02^b^	2.8 ± 1.9^b,c^	7.8 ± 0.4	
		1^st^	O	1.6 ± 0.3^b^	2.03 ± 0.04^b^	2.8 ± 1.9^b,c^		
320°C H_2_/He	Pd-K	1^st^	Ga	1.9 ± 1.1^b^	2.56 ± 0.01^b,c^	10.0 ± 1.9^b,c^	3.3 ± 1.2	4.1
		2^nd^	Pd	8.5 ± 1.8^b^	2.78 ± 0.01^b^	10.5 ± 2.4^b^		
	Ga-K	1^st^	O	3.4 ± 0.7^b^	1.85 ± 0.02^b^	12.9 ± 4.1^b^	2.1 ± 0.4	
		2^nd^	Pd	2.9 ± 0.6^b^	2.58 ± 0.01^b,c^	10.0 ± 1.9^b,c^		
		3^rd^	Ga	4.5 ± 3.0^b^	3.38 ± 0.02^b^	14.8 ± 1.9^b^		
550°C H_2_/He	Pd-K	1^st^	Ga	3.1 ± 0.6^b,d^	2.52 ± 0.01^b,c^	6.5 ± 0.6^b,c^	3.3 ± 1.2	2.5
		2^nd^	Pd	7.5 ± 1.4^b^	2.81 ± 0.02^b^	11.4 ± 2.0^b^		
	Ga-K	1^st^	Pd	5.6 ± 0.3^b^	2.52 ± 0.01^b,c^	6.5 ± 0.6^b,c^	2.1 ± 0.4

The coordination numbers for the Pd atoms differ from the values expected in the bulk structure of GaPd_2_/SiO_2_. The coordination numbers show that Pd in the bulk structure of GaPd_2_/SiO_2_ is, on average, surrounded by 3.5 Ga atoms and by an outer shell of 5.5 Pd atoms. Each Ga atom is surrounded by 10 Pd atoms located in two shells. The first shell is composed of 6 Pd atoms, located around 2.55 Å, whereas the second shell contains 4 Pd atoms located 2.61−2.95 Å from the Ga center. We find that the number of Ga atoms is smaller than expected for a bulk structure, suggesting that the intermetallic catalyst is composed of small particles, supporting the XRD data. Moreover, this also suggests that a significant fraction of Ga atoms are on the surface of the particles, leading to a reduced number of neighbors compared with the bulk. The higher coordination number found for the second shell of Pd in GaPd_2_ is attributed to the combination of the atomic distances of Pd atoms in the GaPd_2_ structure with the expanded atomic distances in Pd hydride which forms in a reducing atmosphere, coming from the 9% unalloyed fraction.

Regarding the chemical environment of Ga atoms, the absence of a second shell in the intermetallic nanoparticle catalyst can be attributed to its longer and broader atomic distances which decreases the amplitude of the EXAFS signal and cause destructive interferences.

**Figure 6.**
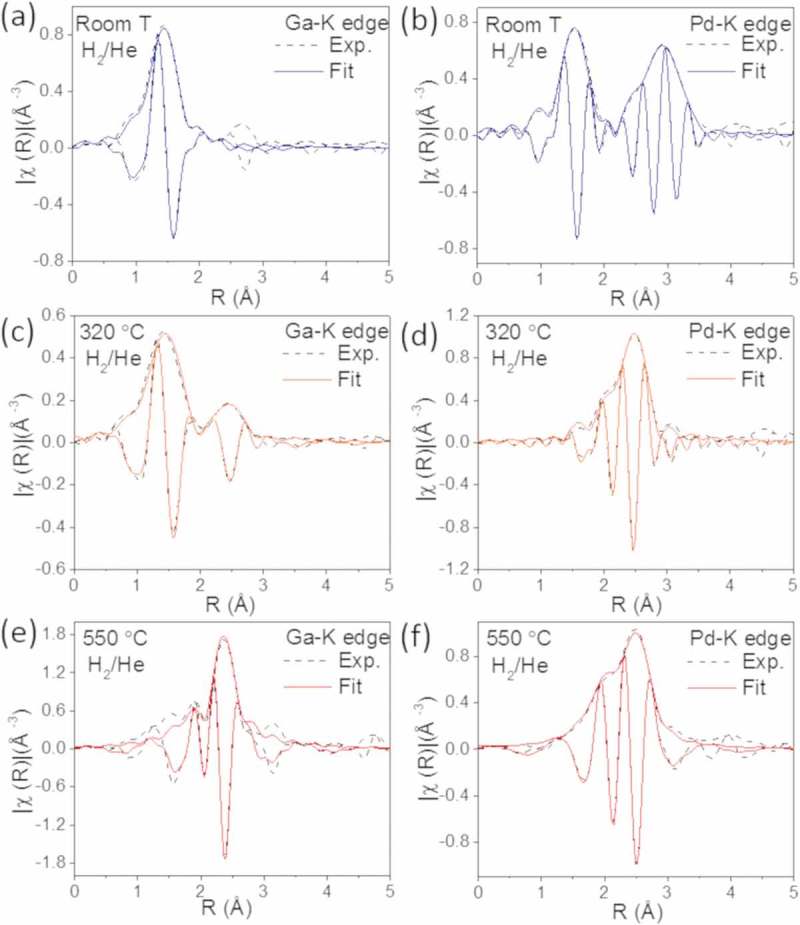
Experimental (dashed lines) and fitted (solid lines) Fourier transformed EXAFS spectra from the catalyst with 13 wt.% metal loading collected in H_2_/He at the Ga-K edge at (a) room temperature, (c) 320°C and (e) 550°C and at the Pd-K edge at (b) room temperature, (d) 320°C and (f) 550°C.

Figure 6 shows the experimental and adjusted Fourier transform of the k^2^-weighted EXAFS spectra collected in a 25% H_2_/He mixture at the Pd-K edge and Ga-K edge from the GaPd_2_/SiO_2_ catalyst with 13 wt.% metal content at three temperatures: 25°C, 320°C and 550°C. The structural parameters obtained from the fitting procedures are presented in Table 2. As for the catalyst with 23 wt.%, the experimental and fitted data for GaPd_2_ crystal structure are in good agreement. Figure 6(a,b) show EXAFS spectra acquired at 25°C in a 25% H_2_/He mixture for the Ga-edge and Pd-edge, respectively. Ga atoms are surrounded by O atoms and, as for the catalyst with 23 wt.% metal loading, the fitting required the inclusion of two O distances, suggesting a distorted oxide structure. The Pd atoms are surrounded by O atoms in the first shell and by Pd atoms in the second shell and third shell. The results indicate that a mixture of PdO and metallic Pd coexist at 25°C in a 25% H_2_/He mixture, in agreement with the *in situ* XRD observations.

**Table 2.** Structural parameters obtained from EXAFS data analysis at Pd and Ga-K edges^a^ acquired from the catalyst with 13 wt.% metal content. Pd S_0_^2^ = 0.83 and Ga S_0_^2^ = 0.88. Pd-Pd atomic distance refined for Pd foil was 2.74 Å. ^b^Adjusted parameter. ^c^Constrained parameter.

13 wt.%	Edge	Shell	Atom	*N*	*R*(Å)	*σ*^2^(10^–3^ Å^2^)	ΔE_0_ (eV)	*ρ* (%)
RT H_2_/He	Pd-K	1^st^	O	3.8 ± 0.3^b^	2.02 ± 0.01^b^	2.5 ± 0.1^b^	1.8 ± 0.2	0.9
		2^nd^	Pd	4.0 ± 0.7^b^	3.05 ± 0.01^b^	1.0 ± 5.**1**^b^		
		3^rd^	Pd	4.0 ± 2.0^b^	3.45 ± 0.02^b^	6.4 ± 3.8^b^		
	Ga-K	1^st^	O	3.6 ± 1.3^b,c^	1.88 ± 0.02^b^	5.2 ± 3.6^b,c^	7.2 ± 0.8	0.2
		1^st^	O	1.4 ± 1.3^b,c^	2.04 ± 0.04^b^	5.2 ± 3.6^b,c^		
320°C H_2_/He	Pd-K	1^st^	Ga	0.9 ± 0.4^b^	2.14 ± 0.01^b,c^	16.5 ± 4.5^b^	4.6 ± 0.6	0.5
		2^nd^	Pd	7.0 ± 0.6^b^	2.74 ± 0.01^b^	7.3 ± 0.6^b^		
	Ga-K	1^st^	O	3.8 ± 0.5^b^	1.89 ± 0.02^b^	10.3 ± 2.3^b^	7.0 ± 1.5	0.3
		2^nd^	Pd	4.3 ± 1.2^b^	2.81 ± 0.03^b^	21.4 ± 3.5^b^		
550°C H_2_/He	Pd-K	1^st^	Ga	3.0 ± 0.9^b^	2.54 ± 0.01^b,c^	7.0 ± 0.7^b,c^	3.7 ± 2.5	1.1
		2^nd^	Pd	7.0 ± 2.4^b^	2.80 ± 0.03^b^	10.6 ± 2.7^b^		
	Ga-K	1^st^	Pd	5.1 ± 0.5^b^	2.53 ± 0.01^b,c^	7.0 ± 0.7^b,c^	1.0 ± 0.7

Figure 6(c,d) show EXAFS spectra acquired at 320°C in a 25% H_2_/He mixture for the Ga-K edge and Pd-K edge, respectively. We find that the Ga atoms have two shells of neighbors, namely O and Pd atoms, while the Pd atoms are surrounded by Ga and Pd atoms. The results are similar to the 23 wt.% results and indicate that at this intermediate temperature a mixture of metallic Pd, GaPd_2_ and gallium oxide coexists. This correlates with XRD analysis, where crystalline GaPd_2_ is observed above 300°C.

Figure 6(e,f) show EXAFS spectra recorded at 550°C in a 25% H_2_/He mixture for the Ga-edge and Pd-edge, respectively. At this temperature, the Ga atoms are only surrounded by Pd atoms and the Pd atoms are surrounded by Pd and Ga atoms, indicating the formation of the GaPd_2_ phase. Also for this catalyst, the coordination numbers for the Pd atoms differ from the values expected in the bulk structure of GaPd_2_/SiO_2_. The alloying is similar for the catalyst with the two metal loadings, but we measure that the coordination numbers are smaller for the 13 wt.% respect to the 23 wt.% sample. This is in line with the XRD results and TEM images, which shows smaller particles for the catalyst with the lower metal content. Moreover, this also suggests that a higher fraction of Ga atoms are on the surface of the particles of the catalyst with the 13 wt.% respect to the 23 wt.% sample. Finally, the lower coordination number found for the second shell of Pd in this catalyst indicates that in the final state the catalyst is phase pure, in contrast to the catalyst with the higher metal loading.

### 3.3 Effect of the reduction temperature on the catalytic activity

**Figure 7.**
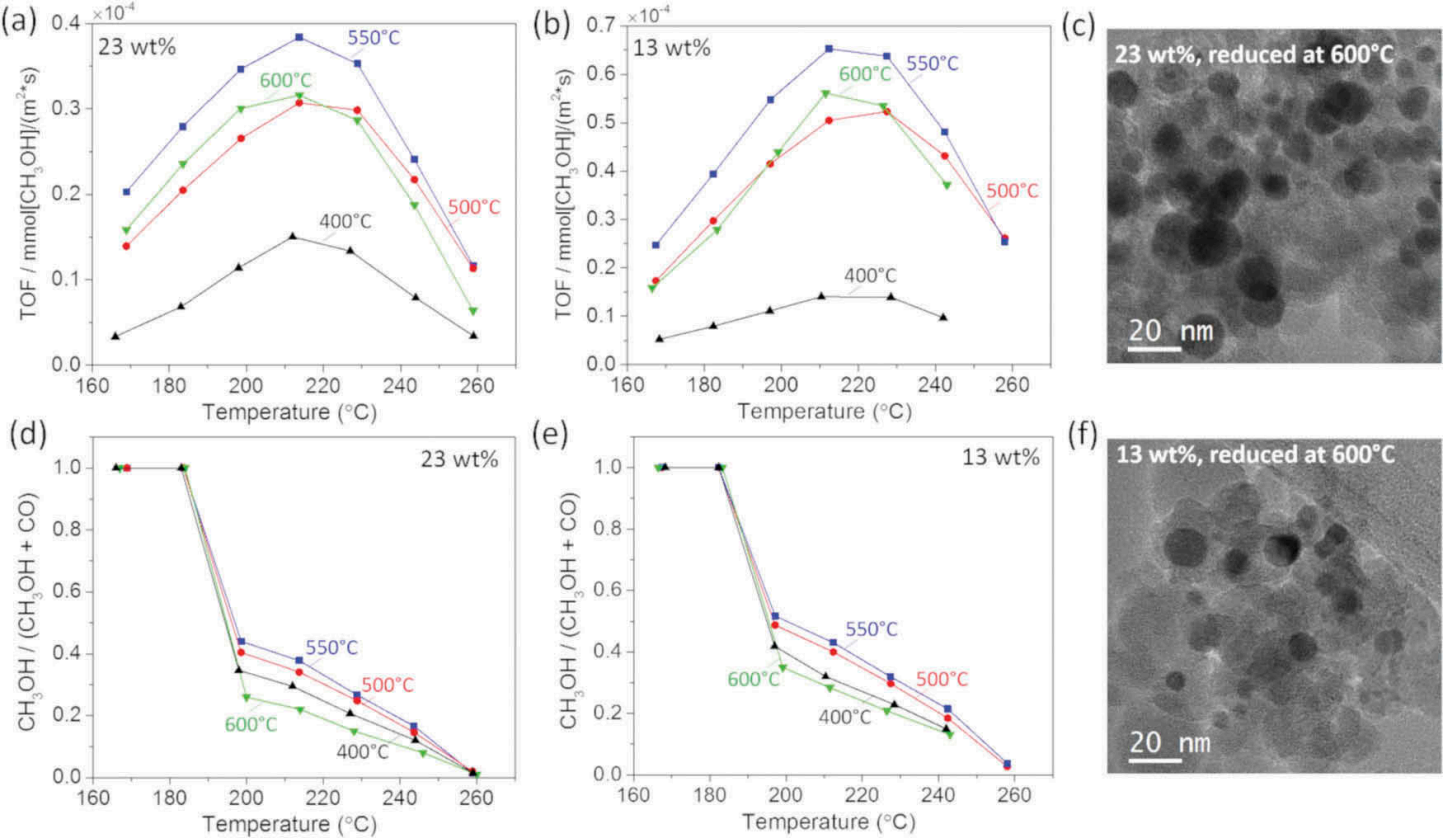
Turnover frequency (TOF) as a function of the reaction temperature measured at four selected reduction temperatures from the GaPd_2_/SiO_2_ catalysts with (a) 23 wt.% and (b) 13 wt.% metal loading. Corresponding CH_3_OH-to-CO ratio from GaPd_2_/SiO_2_ catalyst with (c) 23 wt.% and (d) 13 wt.% metal loading. TEM images of GaPd_2_ nanoparticles with (e) 23 wt.% and (f) 13 wt.% metal loading reduced at 600°C.

From the *in situ* XRD and EXAFS measurements, we learn that the GaPd_2_ is formed at 500°C for both catalysts, however, the palladium is fully reduced at lower temperatures. This encourages us to compare the catalytic activity and the selectivity of the catalyst reduced at four different temperatures, namely 400°C, 500°C, 550°C and 600°C. The TOF and the selectivity of the catalyst with 23 wt.% and 13 wt.% metal loadings are plotted as a function of the reaction temperature in Figure 7(a,b) and (d,e). For both catalysts we find that the TOF and selectivity increases for reduction temperatures up to 550°C and it decreases again at 600°C, indicating that 550°C is the optimal reduction temperature for both catalysts. Figure 7(c,f) shows representative TEM images of the catalyst with 23 wt.% and 13 wt.% metal loadings after reduction at 600°C. By analyzing a collection of similar images we estimated the average particle size as 15 nm and 10 nm for the catalyst with 23 wt.% and 13 wt.% metal loadings, respectively. Smaller average particle sizes are estimated from TEM images of catalysts reduced at lower temperature, as shown in Figure 2. This indicates that the activity loss can be attributed to sintering occurring while reducing the particles at 600°C.

## 4. Discussion

The active phase formation of the GaPd_2_/SiO_2_ catalysts with 23 wt.% and 13 wt.% metal content follows the same pathway, but the 13 wt.% catalyst forms crystalline GaPd_2_ intermetallic nanoparticles at lower temperature than the 23 wt.%. This can be explained considering the size and dispersion of the intermediate Pd particles and of the Ga oxide species in the SiO_2_ support during reduction. The Pd particles are surrounded by the Ga species and they are smaller and higher dispersed in the 13wt.% metal loading catalyst compared to the 23 wt.% metal loading catalyst. This facilitates the diffusion of Ga species into Pd and can explain why the reduction occurs at lower temperature for the smaller particles compared to the larger ones.

The obtained average particle size for the GaPd_2_/SiO_2_ catalyst with 23 wt.% is larger compared to the catalyst with 13 wt.% metal loading. This is a consequence of the sintering of the Pd crystalline particles which takes place during reduction up to 400°C, and also yields a loss in specific surface area of the resulting alloyed catalyst. The results of the catalytic testing of GaPd_2_/SiO_2_ as function of metal loading indicate that both TOF and selectivity depend on the size of GaPd_2_ nanoparticles. An optimum in particle size for the turnover is commonly observed for heterogeneous catalyst [27–31]. Here we find that the optimum particle size of the GaPd_2_/SiO_2_, in terms of catalysts intrinsic activity (TOF), corresponds to an average particle size of 8 ± 2 nm (13 wt.% catalysts). This behavior could indicate that the active sites for methanol production on the surface of GaPd_2_ nanoparticles are neither purely terraces nor lower coordination sites (such as corners and edges), but relies on a combination of both. The lower TOF of smaller nanoparticles is most likely caused by both the CO blocking of edge/corner site and lower intrinsic activity at the small terraces.

Finally, the phase purity differs in the final state of the catalysts with the two metal loadings. The catalyst with the 13 wt.% metal loading is phase pure, whereas the 23 wt.% metal loading catalyst contains 9% of Pd after reduction. Pd is not active for hydrogenation of CO_2_ to methanol [13,32] and its presence lowers the catalytic activity of the 23 wt.% metal loading catalyst. However, the difference in the measured TOF values between the catalysts with 13 wt.% and 23 wt.% cannot be explained solely by the presence of Pd in the catalyst with the higher metal loading: the higher intrinsic activity if the 13 wt.% catalyst is attributed to its phase purity combined with the higher activity of the nanoparticles, with average size of approximately 8 nm.

## 5. Conclusions

In this work, we study the catalytic activity of nanodispersed intermetallic GaPd_2_/SiO_2_ catalysts prepared by incipient wetness impregnation towards the CO_2_ hydrogenation to methanol reaction at ambient pressure, as a function of catalyst metal content. We find that the intrinsic activity is higher for the GaPd_2_/SiO_2_ catalyst with a metal loading of 13 wt.% compared to catalysts with higher or lower metal loading. Moreover, we find that the highest catalytic activity is measured on catalysts reduced at 550°C, above which sintering occurs with consequent loss of active surface area. Based on the results from the catalytic tests, we observe that there is an optimum particle size of the GaPd_2_/SiO_2_ in terms of catalysts intrinsic activity (TOF), corresponding to 8 nm. In order to unravel the formation of the active phase in H_2_ of the calcined GaPd_2_/SiO_2_ catalyst, we use a combination of complementary *in situ* techniques, such as XRD, XANES and EXAFS. The alloying mechanisms are somehow similar for the catalyst with the two metal loadings, but we measure that the coordination numbers from EXAFS are smaller for the 13wt.% respect to the 23wt.% sample. This is in agreement with the XRD results and TEM images, which shows smaller particles for the catalyst with the lower metal content. Moreover, the EXAFS results also indicate two main differences between the catalysts with lower and higher metal contents: compared to the catalyst with the higher metal loading, the surface of the particles of the catalyst with the 13wt.% is more Ga rich and in its final state is phase pure.
